# Occupational exposure to blood and body fluids among primary healthcare workers in Johannesburg health district: High rate of underreporting

**DOI:** 10.4102/safp.v62i1.5027

**Published:** 2020-05-14

**Authors:** Collins C.E. Mbah, Zuberu B. Elabor, Olufemi B. Omole

**Affiliations:** 1 Department of Family Medicine, University of the Witwatersrand, Johannesburg, South Africa; 2 Eastern Cape Department of Health, Aliwal North, South Africa

**Keywords:** blood, body fluids, occupational exposure, healthcare workers, injury reporting, sharps exposure

## Abstract

**Background:**

Healthcare workers (HCWs) are at risk of bloodborne infections from sharp instrument injuries and skin and mucous membrane exposures to contaminated blood and body fluids (BBF). While these have clinical and occupational health implications, little is known about BBF exposure and its reporting pattern in South African primary healthcare (PHC). The aim of this study was to determine the rate of BBF exposure, the extent of reporting and the reasons for not reporting among HCWs in PHC facilities in Johannesburg, South Africa.

**Methods:**

In a cross-sectional study involving 444 participants, an 18-item, self-administered questionnaire was used to collect information on socio-demographic characteristics, HCWs’ exposures to BBF in the last year, whether the exposure was reported and the reasons for not reporting. Analysis included descriptive statistics and chi-square test.

**Results:**

Most participants were nurses (87.4%) and female (88.1%). About a quarter of participants (112) reported having at least one BBF exposure in the last year. Overall, there were 355 exposures, resulting in 0.8 BBF exposure per HCW per year. Of these exposures, 291 (82.0%) were not reported. Common reasons for not reporting include lack of time (42.72%), perception that the source patient was at low risk for human immunodeficiency virus (24.7%) and concerns about confidentiality (22.5%). Blood and body fluids exposures involving nurses (*p* < 0.001), sharp instrument (*p* < 0.001) and HCWs aged < 50 years (*p* = 0.02) were significantly more likely to be reported.

**Conclusion:**

This study found a high rate of underreporting of BBF exposures among HCWs in PHC facilities in Johannesburg, suggesting an urgent need for interventions to improve reporting.

## Introduction

Healthcare workers (HCWs) are at risk of many bloodborne infections following injuries with sharp instruments and exposure of mucous membrane and non-intact skin to contaminated blood and body fluids (BBF). The World Health Organization (WHO) has estimated that 3 million HCWs worldwide experience needlestick and sharps injuries every year.^1^ Auta et al. found the pooled global 1-year prevalence estimate of percutaneous injuries among HCWs to be 36.4%.^2^ In South Africa (SA), the prevalence of percutaneous injuries among HCWs is high with rates as high as 23.35% reported among HCWs at Witbank Hospital.^3^ Also consistent with international reports, doctors and nurses are by far most affected by these injuries.^2^ Although primarily associated with transmission of hepatitis B virus (HBV), hepatitis C virus (HCV) and human immunodeficiency virus (HIV), these injuries and exposures may transmit more than 20 bloodborne pathogens (BBP).^4^ As many as 47% of HBV and 45% of HCV infections in HCWs in the developing world are attributable to percutaneous occupational exposure.^5^ The prevalence of bloodborne virus (BBV) infection in SA HCWs is estimated to mimic that of the general population: HBV 0.2% – 16%, HIV 17.9% and HCV ~2.4%.^6^ Traditionally, the prevention of BBF exposures has been based on the hierarchy of controls, which includes hazard elimination, the use of safer devices, administrative controls involving development of policies on training and educating HCWs on how to limit exposure to BBF, and work practice controls.^7^ In many countries (SA included), it has not been possible to implement preventive strategies because of poor supporting estimates of the burden of occupational exposure to BBP.^8^ This has led to the introduction of the concept of evidence-based prevention (EBP).^9^ Evidence-based prevention involves the use of root cause analysis, a process for identifying causal factors to use in injury prevention. Such information, including the circumstances of occupational transmission of BBP, helps in targeting and evaluating interventions. Reporting injuries and documenting all BBF exposures also enable the employee to receive appropriate post-exposure management and compensation.

Many of the available occupational BBF exposure statistics use data from officially reported incidents that may not be a true reflection of workplace events. Furthermore, studies both locally and internationally have reported high rates of underreporting of BBF exposures among HCWs, with most of these focusing on HCWs at secondary and tertiary healthcare facilities.^10,11,12^ Although the risk of exposure to BBF among HCWs at primary healthcare (PHC) facilities can be extrapolated, to some degree, from the literature on HCWs at secondary and tertiary levels, the rates of reporting or/and underreporting of such exposures, and reasons for underreporting may be very different.

Many PHC facilities in SA have fewer than 10 employees/clinicians, resulting in lack of on-site infection control and employee wellness programmes. Stigma may also be a big factor as the few staff members know each other too well and may not be willing to volunteer personal health information that is required for the reporting of BBF exposure incidents. To this end, little or no data exist on reporting of BBF exposures by HCWs at PHC facilities in SA despite the peculiarities and challenges faced by workers at this service level. We therefore sought to determine the rate of BBF exposure and the reasons for underreporting among HCWs in primary care facilities in Johannesburg.

## Methods

This was a cross-sectional study conducted in 15 primary care clinics, one community health centre and one district hospital in Johannesburg district between January 2013 and April 2013. All surveyed facilities had written protocols or guidelines on reporting of accidental occupational exposures to BBFs by HCWs.

The study population included 544 HCWs made up of 56 medical doctors, four dentists and 484 nurses working in the facilities during the study period. Doctors and nurses who were on leave and all allied HCWs were excluded from this study. Although a sample size of 384 was determined adequate, all the 544 HCWs were invited to participate.

An 18-item self-administered questionnaire adapted from the Centers for Disease Control and Prevention design with demonstrated external validity^13^ was used for data collection. This questionnaire collected data on the socio-demographic characteristics of the participants, their exposures to BBF in the preceding 1 year, whether the exposures were reported or not and their reasons for not reporting, if any.

The first author personally distributed the questionnaires to eligible HCWs at each facility following prior telephonic arrangements with the facility managers. Each facility was visited a minimum of two times for distribution and collection of the questionnaires between January and April 2013. Completed questionnaires were dropped off in a sealed box placed at a known location in each facility. Participation in this study was voluntary and completion of the questionnaire implied consent.

Data were analysed with the help of a statistician using Microsoft Excel spreadsheet for cleaning and coding purposes and Epi Info 7 statistical software (Centers for Disease Control and Prevention in Atlanta, Georgia, United States) for analysis. Outcomes of interest included the number of BBF exposures per participants within the past year, the proportion of exposures reported and the reasons for not reporting, if any.

A pilot study was carried out at a commuinty health centre (CHC) in another sub-district outside the study area to determine the feasibility of the main study. Fifteen respondents (5 doctors and 10 nurses) were included in the pilot study. They were subsequently interrogated by the first author following which minor changes were made to the questionnaires to improve clarity. The results of the pilot study were not included in the final analysis.

### Ethical considerations

Ethical approval for the study was granted by the Human Research Ethics Committee (HREC) of the University of the Witwatersrand (Ethics clearance certificate number M121150). Permission was also granted by the Johannesburg metropolitan health district and the City of Johannesburg local government health department.

## Results

A total of 515 questionnaires were distributed (58 doctors and 457 nurses). Of these, 466 were returned (90.5% response rate). Of the 466 returned, 22 were excluded because of incomplete information, leaving 444 questionnaires (56 doctors and 388 nurses) for analysis.

As shown in Table 1, most of the participants were female (88.1%), nurses (87.4%) and based in the district hospital (54.7%). The mean age was 39.8 years with a mean work experience of 11.7 years.

**TABLE 1:** Participants’ characteristics.

Characteristic	Frequency	%
**Gender**		
Female	391	88.1
Male	53	11.9
**Age groups**		
≤ 30 years	100	22.5
31–40 years	158	35.6
41–50 years	112	25.2
> 50 years	74	16.7
**Occupation**		
Medical doctor/dentist	56	12.6
Enrolled nurse	69	15.5
Enrolled nurse assistant	81	18.3
Professional nurse	238	53.6
**Work experience groups**		
≤ 10 years	257	57.9
11–20 years	105	23.6
21–30 years	54	12.2
> 30 years	28	6.3
**Workplace**		
Clinic	109	24.6
Community health centre	92	20.7
Hospital	243	54.7

A total of 112 participants (25.2%) – 27 doctors and 85 nurses – were exposed to BBF in the last year (Table 2). The total number of exposures was 355, resulting in an exposure rate of 0.8 per HCW per year and three exposures per exposed HCW per year (Table 3). Of the 355 exposures, 159 (44.8%) were caused by sharp and 196 (55.2%) by non-sharp objects. An estimated 82% (291) of the total exposures were not reported to the designated health official.

**TABLE 2:** Frequency of blood and body fluids exposures by professional category.

Profession	Exposed		Unexposed		Total		*p*
	*n* (112)	% (25.2)	*n* (332)	% (74.8)	*N* (444)	% (100)	
**Doctors**	27	48.2	29	51.8	56	100.0	**-**
**All nurses**	85	21.9	303	78.1	388	100.0	< 0.001
**Exposure by nurse category**							
Enrolled nurses	6	8.7	63	91.3	69	100.0	< 0.001
Enrolled nurse assistants	12	14.8	69	85.2	81	100.0	
Professional nurses	67	28.2	171	71.8	238	100.0	

**TABLE 3:** Proportions of exposures reported and unreported.

Type of exposure	Reported		Not reported		*p*	
	*n*	%	*n*	%		
Sharps	50	31.4	109	68.6	< 0.001	
Non-sharps	14	7.1	182	92.9		
**Total**	**64**	**18.0**	**291**	**82.0**	**355**	**100**

Of the 112 HCWs exposed to BBF, 44 (39.3%) reported at least one exposure and only 23 (20.5%) reported all incidents. More than 60% (68) did not report any of the exposures (Table 4).

**TABLE 4:** Reporting patterns.

Participant characteristics	*n*	%
Number of participants exposed to blood and body fluids	112	100.0
Reported all exposures	23	20.5
Reported at least one of the exposures	44	39.3
Did not report any of the exposures	68	60.7

As shown in Table 5, reasons for not reporting BBF exposures included lack of time (42.7%, *n* = 38), perceptions that the source patient was at low risk for HIV (24.7%, *n* = 22) and concerns about confidentiality (22.5%, *n* = 20). Table 6 shows that underreporting of BBF exposures was significantly associated with being > 50 years of age (*p* = 0.018), being a doctor (*p* = 0.011) and having > 20 years of work experience (*p* = 0.049).

**TABLE 5:** Reasons for not reporting exposures across settings and categories.

Reason	Frequency	Clinic (*N* = 20)		CHC (*N* = 13)		Hospital (*N* = 56)		Doctor (*N* = 25)		Nurse (*N* = 64)	
		*n*	%	*n*	%	*n*	%	*n*	%	*n*	%
I did not have time to report	38	11	55.0	5	38.5	22	39.3	14	56.0	24	37.5
I did not know the reporting procedure	14	1	5.0	0	0.0	13	23.2	4	16.0	10	15.6
I was concerned about confidentiality	20	1	5.0	5	38.5	14	25.0	4	16.0	16	25.0
I thought I might be blamed or get in trouble for having the exposure	11	6	30.0	2	15.4	3	5.4	0	0.0	11	17.2
I thought the source patient was low risk for HIV	22	2	10.0	4	30.8	16	28.6	9	36.0	13	20.3
I thought the source patient was low risk for hepatitis B or C	9	2	10.0	3	23.1	4	16.0	4	16.0	5	7.8
I thought the type of exposure was low risk for HIV	19	6	30.0	4	30.8	8	32.0	8	32.0	11	17.2
I thought the type of exposure was low risk for hepatitis B or C	9	3	15.0	3	23.1	4	16.0	4	16.0	5	7.8
I did not think it was important to report	15	4	20.0	2	15.4	6	24.0	6	24.0	9	14.1
I did not want to know my HIV status	1	0	0.0	0	0.0	1	1.8	0	0.0	1	1.6
I did not want staff of this facility to know my HIV status	12	0	0.0	2	15.4	10	17.9	1	4.0	11	17.2
I already knew my HIV status	5	1	5.0	1	7.7	3	5.4	0	0.0	5	7.8

CHC, commuinty health centre; HIV, human immunodeficiency virus.

**TABLE 6:** Association between participants’ descriptors and the number of blood and body fluids reported.

Characteristics	All exposures *N* = 355	Exposures reported		Exposures not reported		*p*
		*n*	%	*n*	%	
**Participant characteristics**						
Female	251	48	19.1	203	80.9	0.40
Male	104	16	15.4	88	84.6	
Age ≤ 50 years	298	60	20.1	238	79.9	0.02
Age > 50 years	57	4	7.0	53	93.0	
**Professional categories and work experience (years)**						
Doctor/dentist	176	22	12.5	154	87.5	0.01
Nurse	179	42	23.5	137	76.5	
≤ 20 years’ work experience	262	54	20.6	208	79.4	0.05
> 20 years’ work experience	93	10	10.7	83	89.3	
**Workplace and OH/EWP training**						
Hospital based	235	47	20.0	188	80.0	0.18
Non-hospital based	120	17	14.1	103	85.9	
Day shift only	139	26	18.7	113	81.3	0.80
Others (night only and day and night)	216	38	17.6	178	82.4	
Had training on infection control	121	28	23.1	93	76.9	0.10
Did not have training on infection control	234	36	15.4	198	84.6	
Had training on OH/EWP	81	17	21.0	64	79.0	0.53
Did not have training on OH/EWP	274	47	17.2	227	82.8	
Familiar with protocol	260	47	18.1	213	81.9	0.37
Not familiar with protocol	30	8	26.7	22	73.3	

OH, occupational health; EWP, employee wellness programme.

## Discussion

This study found that HCWs in this public PHC setting are at significant risk of occupational exposure to BBF. However, most of these incidents were not reported mostly because of lack of time and perceptions that the incident BBF exposure was low risk. Furthermore, doctors and HCWs who were experienced and older were particularly at risk of not reporting these incidents.

Reporting BBF exposures has many values: firstly, it allows the affected HCW to receive appropriate and prompt medical assessment, counselling and treatment and post-exposure prophylaxis – activities that have been shown to reduce the risk of HIV seroconversion by as much as 79%.^11^ Secondly, it enables the award of appropriate compensation as prescribed in the *Compensation for Occupational Injuries and Diseases Act* (COIDA).^14^ Lastly, by documenting exposures, it is possible to identify causes of BBF exposures and assess the effectiveness of preventive measures that have been put in place ensuring EBP.^7^

According to the literature, underreporting of BBF exposures is prevalent and may range between 22% and 82% of incidents.^15,16,17,18^ That 82% of BBF exposures in this study were not reported to a designated official has huge implications for the staff, their families and patients, and negates preventive efforts to minimise BBF exposures. Also, it makes the findings of root cause analysis of the documented exposures unreliable. This high underreporting rate contrasts the finding by Aigbodion et al.^19^ of an injury reporting rate of 98.9% among interns in Johannesburg. It is unclear whether this is because of a lower motivation to maintain health on the part of older HCWs or because of the phenomenon of desensitisation^20^: the more an HCW is exposed to BBFs, the more relaxed they become with regard to reporting the exposures. The latter seems more plausible as it would explain our finding that doctors were significantly more likely to be exposed to, and less likely to report, BBF exposures than nurses.

The findings of this study confirm that lack of time^21^ is one of the most common reported reasons for underreporting of BBF exposures. This may be because of the following:

- The decentralisation and devolution of healthcare services in SA^22^ that has seen the PHC level of care take on more services and resulted in increased workload at PHC facilities. This, coupled with staff shortages and the pressure to push the queue, may cause HCWs to work faster and limit their attention to their own safety procedures.^23^ Overworked HCWs become less alert and more susceptible to BBF exposure^24,25^ and may not have time to report the incidents. This is evident in the high frequency of the reason of ‘lack of time’ among staff working at the clinics (55.0%; Table 5).
- At the time of this study, PHC facilities did not have infection control or employee wellness officer, possibly increasing HCWs’ reluctance to report incidents of BBF exposures. However, each BBF exposure is associated with risk of transmission of diseases (albeit small) if not well managed and this can be devastating for both the HCW and a healthcare system that is under-resourced in terms of human resources.
- Not all PHC clinics in SA have a resident doctor who can do an immediate assessment of staff with BBF exposure and even when present, most are not trained in occupational health assessment, management and administrative processes. The result is that HCWs are expected to report their BBF exposures to their immediate supervisor and then proceed to another facility for assessment and management. In addition, staff members often complain about the lengthy forms they must fill out and the time it takes to get the required signatures of designated officials. These lengthy processes take HCWs away from work for prolonged periods and have negative effects on staff morale, especially in facilities with barely adequate number of staff. The unfriendly processes may result in HCWs being reluctant to report their exposures and with poor management may increase the risk of acquiring and transmitting BBF infections to other patients and their loved ones.

Another common reason for not reporting BBF exposures is perceived low risk of infection transmission.^26^ This perception has been documented among HCWs across several clinical settings^17,18,27^ and represents a great deal of underestimation of risk of infection transmission following BBF exposure. While this study affirmed this, it raises serious clinical and public concerns, especially that SA is endemic for HIV and HBV (13.1% and 4.3%, respectively)^28,29^ and a significant proportion of the disease burden is now handled at the PHC level. Indeed, it may still be possible to transmit bloodborne infections even when the instrument is perceived sterile, for example, in a sharp instrument injury when the HCW is wearing blood-stained gloves. An elderly patient can no longer be considered a low HIV infection risk because of improved medication because HIV/AIDS patients are now living longer. All patients should always therefore be considered potential sources of bloodborne infection and standard universal precautions practised by all HCWs. Although our questionnaire was not designed to determine the circumstances of the respondent’s BBF exposure, the literature is replete with evidence of risk underestimation by HCWs.^19,25,30^ Rapid reporting of needlesticks injuries and splashes leads to a substantial reduction in transmission of BBV infections.^31^ It is imperative that every BBF exposure is reported to enable accurate estimates of the disease burden associated with occupational exposure to BBF and to ensure that EBP strategies are employed.

In this study, 22.5% of the participants did not report their BBF exposures because of concerns about confidentiality and 13.5% did not want other staff members in their facility to know their HIV status. This was more obvious among HCWs in the CHCs than hospitals (38.5% and 15.4% vs. 25.0% and 17.9%, respectively). This may be because staff complements in CHCs are usually smaller and members more closely knit than in the hospitals, making it more difficult to ensure confidentiality. Stigma and concerns about confidentiality have been cited in literature as reasons for underreporting of BBF exposure.^32,33^

More than 17% of nurses reported that they did not report exposures because they thought they might be blamed or get into trouble for having the exposure. The figures are higher among clinic staff members (30.0%) whose productivity is mostly judged by the number of patients they attend to. They may be blamed for not adhering strictly to standard precautions or for being ‘careless’ and as exposure incidents increase in number, these may be used against them during performance evaluation. Thus, HCWs may not be reporting exposures because this would mean abandoning their work, which, in turn, affects their performance evaluation scores. This should not be so, as the socio-economic implications are grave for the HCWs, their dependents and even the employer if they seroconvert – prolonged time off work, expensive medical care and even medical boarding.

The reasons for failing to report a BBF exposure may be multi-factorial and each factor could affect the complex explanation of this undesired behaviour. In the social–cognitive theory of behaviour, Bandura^34^ explains that behaviour, cognition and other personal factors and environmental influences all operate as interacting determinants that influence each other, albeit with different strengths. From this perspective, the final decision of reporting or not depends on the HCWs’ perception of balance of the weights of the factors under the circumstance – if an exposure is perceived low risk but the HCW has plenty of time, he or she is more likely to report the incident than if time is limited. A high-risk exposure may not be reported by a staff member who does not have time or who is very worried about confidentiality. This reciprocal determinism implies that for a successful behavioural change by HCWs regarding exposure reporting, there is a need to recognise the environmental factors that may deter exposure reporting.

A complete overhaul of the implementation of the policy on accidental occupational exposure to BBF is therefore needed in South African PHC. This should align with the findings of Tabak et al.^35^ that the best predictors of compliance to reporting of BBF exposures were the perceived efficiency of the reporting system, perceived severity of acquiring a disease and overall motivation to maintain health. The new system should include the establishment of a centralised, electronic injury reporting system coupled with a 24-h, dedicated local and provincial telephone hotline/helpline to handle BBF exposure reporting. This has been shown to improve reporting compliance significantly.^36^ Healthcare workers could call the helpline from anywhere at any time. The helpline should be linked to a network of personnel trained in injury reporting, counselling and clinical management. These personnel could be readily dispatched to the affected HCWs’ facility to assist them with the necessary support. This will provide consistency and confidentiality and reassure and protect HCWs. It will eliminate the problem of lack of time and increase reporting rates.

There is no doubt that SA has rich experience in the establishment and management of 24-hour helpline as several of these lines are available in the country. However, at this time of economic stagnation and austerity, there is no doubt that budgeting and staffing of the helpline could pose some challenge. Funding for the establishment and running of the helpline could be sourced from the compensation fund. Collaboration between the departments of labour and health will be vital in the development of procedures and guidelines for using the helpline. There is a need to educate HCWs on the usefulness of the helpline for employee buy-in.

Continuous and targeted education of HCWs in the primary care setting on the risks of BBF exposures should be embarked on. This should include training aimed at changing HCWs’ wrong perceptions about the risks of the source of BBF exposure. They should be educated on taking responsibility for their own health and safety and for that of others who may be affected by their actions at work. Awareness on suitability of HBV vaccination should be promoted.^37^ They must be educated on the need to evaluate their long-term risks in terms of possible seroconversion or infection rather than the short-term impact on their work. This could be incorporated into formal infection control training, including the use of universal standard precautions at the workplace which has been shown to improve reporting rate.^38^ Such education and training could be made mandatory for all staff by its inclusion in the performance management and development system.

## Summary

Although BBF exposure rate in this South African PHC setting is high, most incidents were not reported. Reasons for the low rate of BBF exposure reporting include lack of time, low-risk perception of source of BBF and concerns about confidentiality. It is an urgent clinical and occupational health imperative to train HCWs on post-BBF exposure management policies, including preventive measures, objective risk assessment, the importance and mechanism of reporting and post-exposure drug treatment guidelines.^17,24^

## Limitations

This study has some potential limitations: It relied on self-reports and is therefore susceptible to recall and information bias. However, BBF exposure is a significant event that is unlikely to be easily forgotten, especially that the period under consideration was limited to incidents that occurred in the last 12 months. The cross-sectional design precludes causal links, and attempts to generalise the findings need to take into account that this study was limited to only one district. Nonetheless, this study is one of few that have investigated exposure and reporting of BBF in South African primary care and highlights the need for interventions to promote reporting of every incident in this setting.

## Conclusion

Accidental occupational exposure to BBF is common but under-reported in this typical South African PHC setting. Interventions aimed at addressing the low rate of reporting need to take cognisance of reasons such as lack of time, lack of confidentiality and low-risk perceptions. To upscale reporting, HCWs need to be trained on objective risk assessment, appropriate clinical response and prompt reporting procedures after BBF exposures. The establishment of a confidential 24-h central telephone hotline for reporting BBF exposures may facilitate reporting by eliminating some of the administrative barriers to reporting.

## References

[1] World Health Organization. The world health report 2002: Reducing risks, promoting healthy life [homepage on the Internet]. [cited 2012 May 15]. Available from: http://www.who.int/whr/2002/en/whr02_en.pdf

[2] Auta A, Adewuyi EO, Tor-Anyiin A, et al. Global prevalence of percutaneous injuries among healthcare workers: A systematic review and meta-analysis. Int J Epidemiol. 2018;47(6):1972–1980. 10.1093/ije/dyy208 30272173

[3] Lachowicz R, Matthews PA. The pattern of sharps injury to healthcare workers at Witbank hospital. SA Fam Pract. 2009;51(2):148–151. 10.1080/20786204.2009.10873831

[4] Centers for Disease Control and Prevention. Workbook for designing, implementing, and evaluating a sharps Injury prevention program [homepage on the Internet]. [cited 2012 May 15]. Available from: www.cdc.gov/sharpssafety/pdf/sharpsworkbook_2008.pdf

[5] Pruss-Ustun A, Rapiti E, Hutin Y. Estimation of the global burden of disease attributable to contaminated sharps injuries among health-care workers. Am J Ind Med. 2005;48(6):482–490. 10.1002/ajim.20230 16299710

[6] Rossouw TM, Van Rooyen M, Louw JM, Richter KL. Blood-borne infections in healthcare workers in South Africa. SAMJ. 2014;104(11):732–735. 10.7196/SAMJ.8518 25909108

[7] Wilburn SQ, Ejikemans G. Preventing Needlestick injuries among health care workers: A WHO-ICN collaboration. Int J Occup Environ Health. 2004;10(4):451–456. 10.1179/oeh.2004.10.4.451 15702761

[8] Pruss-Ustun A, Rapiti E, Hutin Y. Sharps injuries: Global burden of disease from sharps injuries to health-care workers. WHO Environmental Burden of Disease Series, No. 3. Geneva: World Health Organization; 2003.

[9] Wilburn SQ. Needlestick and sharps injury prevention. Online J Issues Nurs. 2004;9(3):5. 15482091

[10] Zungu LI, Sengane ML, Setswe KG. Knowledge and experiences of needle prick injuries among nursing students at a university in Gauteng, South Africa. SA Fam Pract. 2008;50(5):48. 10.1080/20786204.2008.10873762

[11] Karani H, Rangiah S, Ross AJ. Occupational exposure to blood-borne or body fluid pathogens among medical interns at Addington Hospital, Durban. S Afr Fam Pract. 2011;53(5):462–466. 10.1080/20786204.2011.10874135

[12] Karstaedt AS, Pantanowitz L. Occupational exposure of interns to blood in an area of high HIV seroprevalence. SAMJ. 2001;91(1):57–60. 11236300

[13] Fullerton M, Gibbons V. Needlestick injuries in a healthcare setting in New Zealand. N Z Med J. 2011;124(1335):33–39. 21946680

[14] Republic of South Africa. The Compensation for Occupational Injuries and Diseases Act, No 130 of 1993 (COIDA). Department of Labour, South Africa.

[15] Tarantola A, Abiteboul D, Rachline A. Infection risks following accidental exposure to blood or body fluids in health care workers: A review of pathogens transmitted in published cases. Am J Infect Control. 2006;34(6):367–375. 10.1016/j.ajic.2004.11.011 16877106 PMC7115312

[16] Schmid K, Schwager C, Drexler H. Needlestick injuries and other occupational exposures to body fluids amongst employees and medical students of a German university: Incidence and follow up. J Hosp Infect. 2007;65(2):124–130. 10.1016/j.jhin.2006.10.002 17174445

[17] Doebbeling BN, Vaughan TE, McCoy KD, et al. Percutaneous injury, blood exposure, and adherence to standard precautions: Are hospital based health care providers still at risk? Clin Infect Dis. 2003;37(8):1006–1013. 10.1086/377535 14523763

[18] Awawu GN, Kabir S, Istifanus AJ. Occupational exposure to blood and body fluids among primary health-care workers in Kaduna State, Nigeria. J Med Trop. 2016;18(2):79–85. 10.4103/2276-7096.192223

[19] Aigbodion SJ, Motara F, Laher AE. Occupational blood and body fluid exposures and human immunodeficiency virus post-exposure prophylaxis amongst intern doctors. S Afr J HIV Med. 2019;20(1):958. 10.4102/sajhivmed.v20i1.958 PMC6561641 31205779

[20] Makary MA, Attar AA, Holzmueller CG, et al. Needlestick injuries among surgeons in training. N Engl J Med. 2007;356(26):2693–2699. 10.1056/NEJMoa070378 17596603

[21] Burke S, Madan I. Contamination incidents among doctors and midwives: Reasons for nonreporting and knowledge of risks. Occup Med. 1997;47(6):357–360. 10.1093/occmed/47.6.357 9327639

[22] Hendricks SJH, Buch E, Seekoe E, Bossert T, Roberts M. Decentralisation in South Africa: Options for district health authorities in South Africa. SAHR. 2014/15;4:59–72.

[23] Patrician PA, Pryor E, Fridman M, Loan L. Needlestick injuries among nursing staff: Association with shift-level staffing. Am J Infect Control. 2011;39(6):477–482. 10.1016/j.ajic.2010.10.017 21612843

[24] Clarke SP, Sloane DM, Aiken LH. Effects of hospital staffing and organizational climate on needlestick injuries to nurses. Am J Public Health. 2002;92(7):1115–1119. 10.2105/AJPH.92.7.1115 12084694 PMC1447200

[25] Auta A, Adewuyi EO, Tor-Anyiin A, et al. Healthcare workers’ occupational exposures to body fluids in 21 countries in Africa: Systematic review and meta-analysis. Bull World Health Organ. 2017;95(12):831–841F. 10.2471/BLT.17.195735 29200524 PMC5710084

[26] Haiduven DJ, Simpkins SM, Phillips ES, Stevens DA. A survey of percutaneous/mucocutaneous injury reporting in a public teaching hospital. J Hosp Infect. 1999;41(2):151–154. 10.1016/S0195-6701(99)90053-1 10063478

[27] Diprose P, Deakin CD, Smedley J. Ignorance of post exposure prophylaxis guidelines following HIV needle-stick injury may increase the risk of seroconversion. Br J Anaesth. 2000;84(6):767–770. 10.1093/oxfordjournals.bja.a013591 10895754

[28] STATS SA. Statistical Release P0302 Mid-year population estimates 2018 [homepage on the Internet]. [cited 2018 Jan 21]. Available from: www.statssa.gov.za/publication/p0302/p0302218.pdf

[29] Spearman CWN, Sonderup MW. Preventing hepatitis B and hepatocellular carcinoma in South Africa: The case for a birth-dose vaccine. S Afr Med J. 2014;104(9):610–612. 10.7196/SAMJ.8607 25212400

[30] Patterson JM, Novak CB, Mackinnon SE, Patterson GA. Surgeons’ concern and practices of protection against bloodborne pathogens. Ann Surg. 1998;228(2):266–272. 10.1097/00000658-199808000-00017 9712573 PMC1191469

[31] Ebrahimi MH, Mirrezaie SM, Aghayan SS, et al. Occupational exposure to blood and other bodily fluids among laboratory technicians: An underestimated risk factor. Int J Health Stud. 2015;1(1):24–27.

[32] Kerr HL, Stewart N, Pace A, Elsayed S. Sharps injury reporting amongst surgeons. Ann R Coll Surg Engl. 2009;91(5):430–432. 10.1308/003588409X432194 19622260 PMC2758447

[33] Yoshikawa T, Wada K, Lee JJ, et al. Incidence rate of needle-stick and sharps injuries in 67 Japanese hospitals: A national surveillance study. PLoS One [serial online]. 2013 [cited 2013 Nov 24];8(10):3. Available from: www.plosone.org 10.1371/journal.pone.0077524 PMC3813677 24204856

[34] Bandura, A. Social cognitive theory. In: Vasta R, editor. Annals of child development. Vol. 6. Six theories of child development. Greenwich, CT: JAI Press, 1989; p. 1–60.

[35] Tabak N, Shiaabana MA, Shasha S. The health beliefs of hospital staff and the reporting of needlestick injury. J Clin Nurs. 2006;15(10):1228–1239. 10.1111/j.1365-2702.2006.01423.x 16968427

[36] HSE reporting guidance [homepage on the Internet]. [cited 2019 Sep 10]. Available from: http://www.hse.gov.uk/riddor/index.htm; http://www.hse.gov.uk/toolbox/managing/reporting.htm

[37] Sali SH, Merza MA, Yadegarina D. Occupational exposure to blood borne viruses among healthcare workers in a tertiary care referral hospital in Tehran. Hepat Mon [serial online]. 2013[cited 2014 Jan 3];13(7):e12201. Available from: www.ncbi.nlm.nih.gov/pmc/articles/pmc3776233 10.5812/hepatmon.12201 PMC3776233 24065999

[38] Ersin F, Koruk ST, Yilmaz L. Effect of the training provided for nurses on sharp – Needlestick injuries and reporting process. Int J Caring Sci. 2016;9(2):561.

